# Effect of differentiated service delivery models for HIV treatment on healthcare providers’ job satisfaction and workloads in sub-Saharan Africa: a mixed methods study from Malawi, Zambia, and South Africa

**DOI:** 10.1186/s12960-025-00993-6

**Published:** 2025-05-26

**Authors:** Vinolia Ntjikelane, Bevis Phiri, Jeanette L. Kaiser, Sydney Rosen, Allison J. Morgan, Amy Huber, Idah Mokhele, Timothy Tchereni, Stanley Ngoma, Priscilla Lumano-Mulenga, Sophie Pascoe, Nancy Scott

**Affiliations:** 1 Health Economics and Epidemiology Research Office, Faculty of Health Sciences, University of the Witwatersrand, Sunnyside Office Park, 32 Princess Wales Terrace, Parktown, Johannesburg, 2193 South Africa; 2 Clinton Health Access Initiative-Zambia, Lusaka, Zambia; 3 School of Public Health, Boston University, Boston, USA; 4 Clinton Health Access Initiative-Malawi, Lilongwe, Malawi; 5 Ministry of Health, Lilongwe, Malawi; 6 Ministry of Health, Lusaka, Zambia

**Keywords:** Differentiated service delivery, HIV, Job satisfaction, Treatment providers, South Africa, Malawi, Zambia

## Abstract

**Introduction:**

HIV care providers are often overworked and suffer from burnout and low job satisfaction. Differentiated service delivery (DSD) models for HIV treatment aim to decongest clinics and improve providers’ quality of professional life by reducing the client/provider ratio and allowing for more time with clients in need. We investigated current job satisfaction and perceived changes in job satisfaction among HIV care providers in Malawi, South Africa, and Zambia after the adoption of DSD models of care in each country.

**Methods:**

We conducted a concurrent, cross-sectional mixed methods survey with clinical and non-clinical HIV care providers between April 2021 and January 2022 at public sector clinics in Malawi (*n* = 12), South Africa (*n* = 21), and Zambia (*n* = 12). Questions investigated the effect of DSD models on provider responsibilities, work burden, time allocation, and job satisfaction. We conducted a principal components analysis of survey responses to create a job satisfaction index and estimated odds ratios (OR) using logistic regression for associations between key variables and low reported job satisfaction. We reported emerging qualitative themes. We used Herzberg’s two-factor theory to organize and interpret results, identifying motivating factors (which lead to job satisfaction) and hygiene factors (which we refer to as maintenance factors, that lead to dissatisfaction if lacking).

**Results:**

Providers had generally high job satisfaction. Providers from Malawi were more likely to report lower job satisfaction than those from South Africa or Zambia (adjusted OR (aOR) 4.56; 95% confidence interval (CI) [2.12–9.80]). Providers who believed that their jobs became harder after the introduction of DSD models (2.82; [1.14–6.96]) or that their jobs did not change (6.50; [2.50–16.89]) were more likely to report lower job satisfaction than those who believed their jobs became easier. Qualitatively, providers felt DSD models improved their working conditions by easing clinic congestion and allowing them to spend more time on other tasks. Providers were particularly motivated when they could spend more time with clients.

**Conclusion:**

Findings highlight the importance of DSD models in maintaining or improving healthcare providers' quality of professional life and underscore the need for continued monitoring of the impact of these models on job satisfaction among HIV care providers in resource-constrained settings.

**Supplementary Information:**

The online version contains supplementary material available at 10.1186/s12960-025-00993-6.

## Introduction

In an effort to improve treatment outcomes for people living with HIV and make service delivery more efficient, many countries in sub-Saharan Africa (SSA) have scaled up “differentiated service delivery” (DSD) models for HIV treatment [1, 2]. DSD is a client-centred approach that is intended to simplify and adapt HIV services to better meet the needs of people living with HIV and reduce the burden of HIV care on the healthcare system, providers, and clients [3]. Common DSD models include community-based medication pickup points, which make access to medications more convenient and less time-consuming; adherence clubs that provide both treatment and social support to groups of clients; and multi-month dispensing of medications to reduce the required number of clinic visits per year. Most of these models require that clients have at least 6 months’ experience on antiretroviral therapy (ART) and documentation of a suppressed viral load [4]. DSD models' design, implementation, and clinical outcomes have been widely documented in recent years [5–10].

Previous studies have defined provider job satisfaction [11] as ‘a gratifying and positive emotional state from the appraisal of one’s job or experiences’ [12]. It is critical to the delivery of high-quality care because it fosters positive provider–client relationships, leading in turn to improved communication, trust, client satisfaction, adherence to medication, and scheduled appointment compliance [13]. A 2018 review of systematic reviews found that client-centred care interventions can improve provider job satisfaction. DSD models, which are a client-centred strategy, may thus also increase HIV treatment providers’ job satisfaction [11]. DSD models have the potential to achieve this—though have not yet been proven to do so—by lightening providers’ workloads and reducing the ratio of client visits per provider, thereby allowing providers either to spend more time with HIV clients who are not eligible for DSD models [12] or to invest more time in other responsibilities, such as care for clients with other conditions or administrative duties. Whether they achieve this goal has not been well documented. Some previous studies have examined healthcare providers’ job satisfaction in sub-Saharan Africa in general [14–17], but none have looked at how DSD implementation affects HIV treatment providers’ satisfaction or the factors causing it.

Herzberg’s two-factor theory, also known as the motivator–hygiene theory, proposes that two distinct and parallel sets of factors (those that prevent dissatisfaction and those that promote satisfaction and engagement) contribute to job satisfaction in health care settings and elsewhere [18, 19]. It suggests that different strategies may be needed to address dissatisfaction and foster motivation to ensure a satisfied workforce and reduce healthcare worker burnout or turnover. Guided by Herzberg ‘s two-factor theory, we use mixed methods to systematically investigate current job satisfaction and perceived changes in job satisfaction before and after DSD implementation among HIV treatment providers in Malawi, South Africa, and Zambia.

## Methods

### Study design and setting

The AMBIT Project’s SENTINEL study is a repeated, cross-sectional, mixed methods survey of differentiated service delivery for HIV treatment at public sector clinics in Malawi, South Africa, and Zambia [26]. SENTINEL includes surveys of ART clients, HIV testers, and treatment providers. Survey instruments concurrently collect both quantitative and qualitative information from the participants. Here we report results from the SENTINEL round 1 provider survey conducted between April 2021 and January 2022.

SENTINEL study sites include 12 public sector clinics in Malawi, 21 in South Africa, and 12 in Zambia. Study sites were purposively selected to provide: 1) sufficient ART client volumes to capture provider experiences in busy resource-constrained environments; 2) variation in settings (rural or urban) to allow for assessment of how DSD implementation varies by setting; and 3) experience implementing DSD models for HIV treatment to ensure that providers had meaningful DSD model exposure. Additional information on study sites is provided in Supplementary Table 1 and in the published protocol [27]. DSD models were incorporated into national HIV treatment guidelines in 2018 in Malawi, 2016 in South Africa, and 2017 in Zambia. There were initially a large number of models implemented, each designed slightly differently and often with the support of nongovernmental partners and external funders [8]. As countries, implementing partners, and facilities gained experience with DSD, a smaller number of national models were scaled up in each country, with a few bespoke, population-specific models remaining alongside them. At the time of data collection for this study, the most commonly implemented models were six-month medication dispensing (6MMD) and mother–infant pairs in Malawi; facility-based pickup and external pickup points in South Africa; and 6MMD and community ART access points (CAAPs) in Zambia (Table 1).

**Table 1:** Models of care commonly offered at study sites

Model	Description	# of expected facility visits and other provider interactions per year in DSD guidelines at time of data collection
Malawi		
Conventional care	Clients receive a 3-month supply of medications at each full clinic visit	4 facility visits; 0 other interactions
Six-month medication dispensing (6MMD)	Clients receive a 6-month supply of antiretroviral medications at each full clinic visit	3 facility visits*; 0 other interactions
Mother–infant pair	The post-partum visits for the mother are aligned to the infant visit schedule. Infant’s schedule is based on the vaccination milestones. Thereafter the mother receives a 3-month supply of antiretroviral medications at each visit	4 facility visits**; 0 other interactions
South Africa		
Conventional care	Clients receive a 2-month supply of medications at each full clinic visit	6 facility visits; 0 other interactions
Facility-based pickup points	Between full clinic visits, clients pick up medications (usually a 2-month supply) at specified pickup points in facilities	2 facility visits; 4 medication pickups
External pickup points	Between full clinic visits, clients pick up medications (usually a 2-month supply) at specified pickup points in the community (e.g. commercial pharmacy)	2 facility visits; 4 medication pickups
Zambia		
Conventional care	Clients receive a 3-month supply of medications at each full clinic visit	4 facility visits; 0 other interactions
Six-month medication dispensing (6MMD)	Clients receive a 6-month supply of antiretroviral medications at each full clinic visit	3 facility visits; 0 other interactions
Community ART access points (CAAPs)	A lay worker collects 3-month supply of medication for 8 clients and distributes it at a designated CAAP	2 facility visits; 2 other interactions

*The third visit is for viral load review

** Visits are not uniform; timing varies depending on the infant’s age

#### Theoretical framework

Herzberg’s two-factor theory of job satisfaction guided our analysis and interpretation [18, 19]. Hygiene or maintenance factors are factors that, if lacking, can cause dissatisfaction among employees, but their presence alone does not necessarily lead to satisfaction. Examples include job security, salary, and working conditions. Motivating factors are those that, when present, can lead to improved job satisfaction and motivate employees to perform at a higher level. Examples include achievement, recognition, responsibility, advancement, and the work itself.

Fundamentally, Herzberg’s theory suggests that satisfaction and dissatisfaction are on different continuums and influenced by different factors, with maintenance factors preventing dissatisfaction and motivating factors driving satisfaction and motivation. Herzberg’s model presumes that individuals often experience motivators and maintenance factors simultaneously, and that these factors are independent of one another (i.e. motivators cannot increase or decrease dissatisfaction; they can only influence the degree of satisfaction). Understanding that an employee may at once be satisfied and dissatisfied is important to explaining employees’ perspectives. Herzberg’s theory has been widely applied in the healthcare, hospitality and tourism [20], utilities, services, retail, manufacturing industries [21], among others, and within varying cultural contexts, including Jordan [22], Sweden [20, 23], Saudi Arabia [24], and the United Kingdom [21]. A recent systematic review has utilized Herzberg’s factors to frame findings of job satisfaction among primary healthcare providers across the world [25].

We use this theory to organize and interpret the results of this study. Throughout, we consider any response that allows providers to spend more time with clients as a Motivating Factor (work itself, time spent with clients) and time for all other tasks as a Maintenance Factor (working conditions, time for other tasks) (Table 2).

**Table 2:** Herzberg’s two-factor theory and its application to our analysis

Variable	Definition	Herzberg theory factor examples (applications to our study)
Maintenance factors (Hygiene)	Necessary to not be dissatisfied, but do not necessarily lead to satisfaction	Job security Working conditions (e.g. time for tasks, burn out from overwork) Compensation
Motivating factors (Motivators)	When present, can lead to improved job satisfaction	Achievement Recognition (e.g. meeting DSD targets) Responsibility Advancement (e.g. training opportunities) Work itself (e.g. intrinsic motivation from relationships with clients, time predictability)

### Participants and data collection

At each study site, facility operations managers referred to the study up to 10 healthcare workers who had been employed at the facility for ≥ 6 months and directly or indirectly involved in the implementation of DSD models. Potential survey participants included facility operations managers, nurses, lay counsellors, community health workers (CHWs), pharmacists, pharmacy assistants, and other cadres involved in DSD for HIV treatment. Study staff introduced the study, conducted the written informed consent process, and then administered the survey to the participants individually in a private area at the facility during a time that was convenient for the respondent.

Data were captured on tablets. Responses to qualitative and open-ended questions were typed verbatim into the tablet by the data collector.

### Measurements

The survey instrument (supplementary file 1) included open- and closed-ended questions pertaining to providers’ experiences with DSD model implementation. Questions included on the survey were guided by a review of the literature and existing data gaps, experience of the research team in previous exploring aspects related to DSD, and priority research questions identified through consultation with stakeholders [8, 10]. The survey questions encompassed provider involvement in DSD models offered at the facility, providers’ opinions regarding DSD models, and the challenges they faced in implementing them, as well as how DSD models affected their job responsibilities, time allocation, and job satisfaction. All participants were asked about their current experience delivering care with the DSD models; participants who had been engaged in service delivery before DSD models were available were also asked about changes they had observed since DSD was introduced.

Survey participants responded to 7 statements that were developed by the study team on the effect of DSD models on provider job satisfaction. These questions used a 5-point Likert scale with response options of strongly disagree, mildly disagree, neither agree nor disagree, mildly agree and strongly agree. The statements assessed participants’ job satisfaction, changes in workload, work schedules, client interactions, relationships with other colleagues and senior management, and relationships with clients, along with overall happiness and commitment to their work since the facilities began offering DSD models.

### Quantitative analysis

We first generated descriptive statistics for participants’ work characteristics, their roles and involvement in DSD models, their views on the effect of DSD models on their job responsibilities and job changes after DSD implementation. We then conducted a principal component analysis (PCA) to create an index for job satisfaction from the 7 Likert scale questions. The final scale’s reliability was determined using a Cronbach's alpha coefficient of 0.60, with factors loading above 0.60 were retained for subsequent regression analyses. We categorized the final mean satisfaction scores as "high" satisfaction (score > 4) or "low" satisfaction (score ≤ 4) in order to simplify interpretation. Finally, we used logistic regression to examine univariate and adjusted associations between key predictor variables and low reported job satisfaction. Significance was considered at p < 0.05, confidence intervals (CI) of 95%.

### Qualitative analysis

For qualitative responses, a codebook was developed inductively for each question by reading through at least 60% of responses. Codes were developed and refined and concepts collapsed or separated based on the content of the responses as coding proceeded. Once a codebook was finalized, each question was reread and assigned all relevant codes in Excel, and additional codes were added if they arose from the data. During analysis, codes were compared by country and provider cadre (clinical vs. non-clinical); those with the highest volumes were identified as major themes in the data. Notable divergent views were identified and reviewed by the study team to reach concurrence. Results were summarized and quotes were identified within each stratum as illustrative examples. Some quotations were edited slightly for grammar and clarity. Results were triangulated with quantitative findings and interpreted within the Herzberg two-factor theory.

## Results

### Participant characteristics and work responsibilities

We enrolled a total of 468 providers: 142 from Malawi, 206 from South Africa, and 120 from Zambia. Nurses comprised the largest cadre in the study (40% of providers), followed by lay counsellors and community health workers (CHWs) (26%), and administrators and data clerks (13%) (Table 3).

**Table 3:** Work characteristics of survey participants

Characteristic*	Total		Malawi		South Africa		Zambia	
	All providers	Provided care pre-DSD*	All providers	Provided care pre- DSD	All providers	Provided care pre-DSD	All providers	Provided care pre-DSD
N	468	364	142	136	206	158	120	70
Cadre, n (%)								
*Doctor, clinical officer, or medical officer*	53 (11%)	44 (12%)	36 (25%)	34 (25%)	1 (0%)	1 (1%)	16 (13%)	9 (13%)
*Nurse*	187 (40%)	154 (42%)	49 (35%)	47 (35%)	111 (54%)	91 (58%)	27 (23%)	16 (23%)
*Pharmacist or assistant pharmacist*	30 (6%)	17 (5%)	1 (1%)	1 (0%)	18 (9%)	14 (9%)	11 (9%)	2 (3%)
*Laboratory staff*	15 (3%)	13 (3%)	12 (8%)	11 (8%)	0 (0%)	0 (0%)	3 (3%)	2 (3%)
*Administrative clerk or data capturer*	60 (13%)	35 (10%)	13 (9%)	12 (9%)	29 (14%)	15 (9%)	18 (15%)	8 (11%)
*Lay counsellor or community healthcare worker*	123 (26%)	101 (28%)	31 (22%)	31 (23%)	47 (23%)	37 (23%)	45 (38%)	33 (47%)
Years’ work experience, median (IQR)	7 (3–11)	8 (4, 12)	6 (3–10)	8 (4, 12)	8 (5–12)	8 (4, 12)	4 (2–10)	7 (3, 11)
Years at facility, median (IQR)	4 (2–9)	5 (3, 10)	4 (2–8)	5 (2, 10)	5 (2–9)	5 (2, 10)	3 (1–9)	5 (2, 9)
Reported direct involvement with DSD implementation	389 (83%)	313 (86%)	142 (100%)	136 (100%)	128 (62%)	108 (68%)	119 (99%)	69 (99%)
Reported that DSD models have affected job, such as responsibilities, work load, hours, n (%)***	–	277 (76%)	–	107 (79%)	–	105 (66%)	–	65 (93%)
No change perceived after DSD implementation, n (%)***	–	87 (24%)	–	29 (21%)	–	53 (34%)	–	5 (7%)

*Age and sex of respondents were not collected

**Characteristics of providers who reported providing HIV care at a time when other models of treatment delivery were not being offered?

**Percentages refer to the proportion of providers who provided HIV care at current facility prior to DSD implementation

Of the 468 participants enrolled, 364 (78%) had provided HIV care before DSD scale-up and could therefore offer a comparison of their experience before and after DSD implementation. Table 3 reports characteristics for this subset as well as for each country’s sample as a whole.

#### Provider workloads and job responsibilities

**Fig. 1:**
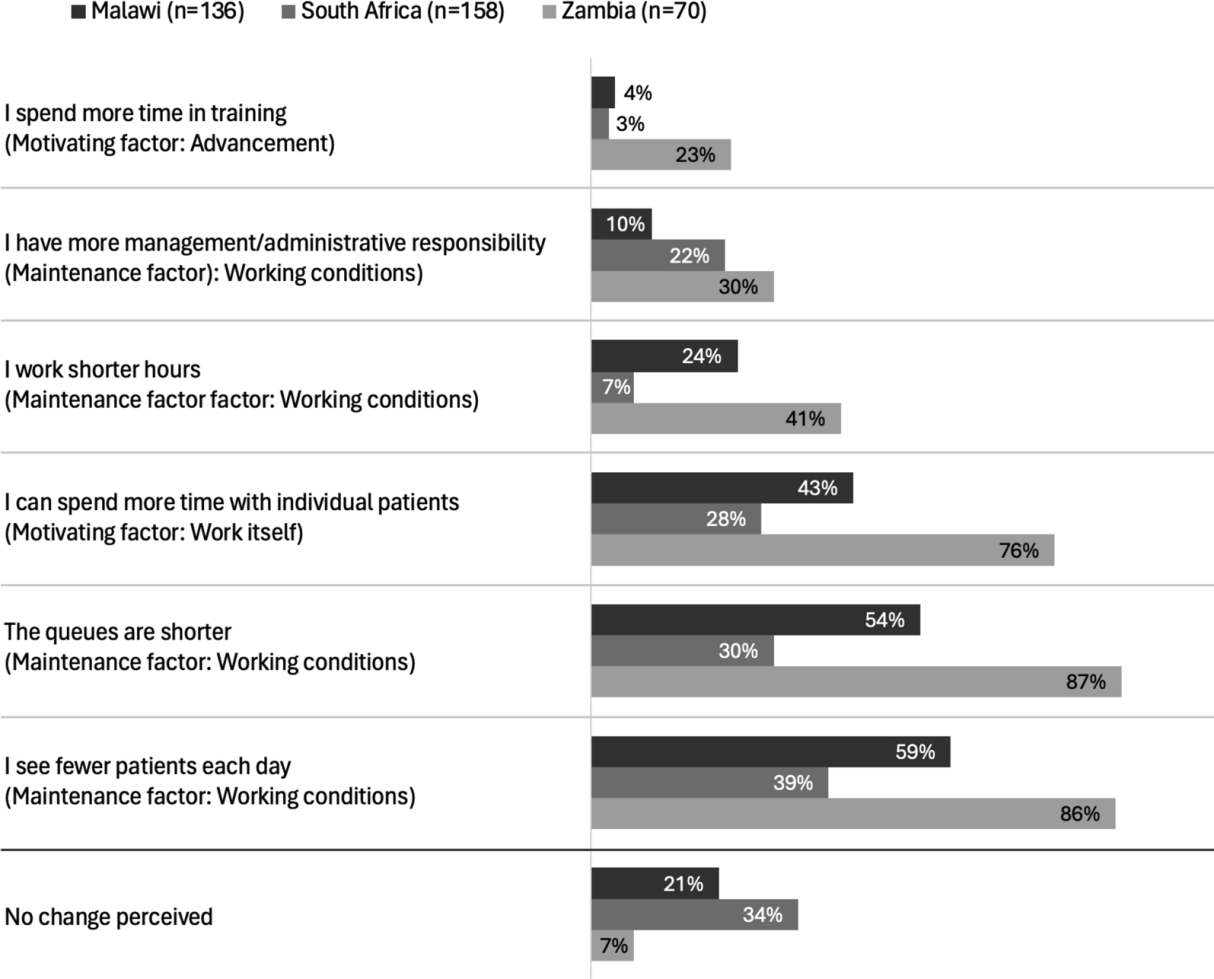
Reported job changes after DSD implementation among providers who provided HIV care at current facility prior to DSD implementation in Malawi, South Africa, and Zambia (*n* = 364)

Among the subset of providers who could compare experiences before and after DSD implementation (*n* = 364), most providers reported that DSD models led to shorter queues, seeing fewer clients per day, and increased time spent with individual clients, especially among providers in Zambia (Fig. 1; Supplementary Table 3 for results by cadre).

Qualitatively, respondents in all three counties overwhelmingly reported reduced workloads as a result of DSD implementation (Table 4). They described the clinics as being "less congested" due to the DSD models diverting clients to external dispensing points, dispensing multiple months of medication at once so that clients make fewer visits to the facility, and/or the management of stable ART clients in a dedicated DSD space, separate from unstable or complex clients with comorbidities. Respondents felt that clinic decongestion, a maintenance factor, led to shorter queues of clients, meaning that providers see fewer clients daily, thereby reducing their workload and giving them more time to allocate to other maintenance and motivating factors. Respondents indicated that they are now able to spend more time with clients, which has led to better relationships with the clients. They also reported being able to allocate time to administrative tasks indicating that they felt the work was being completed properly (Table 4).

**Table 4:** Healthcare worker perspectives on how DSD implementation has affected their workload and time spent at work from Malawi, South Africa and Zambia

Effects on time spent at work	Illustrative quotes
Spend more time with clients (Motivating factor: work itself)	*“…[The] number of clients that visit the facility has reduced as a result of models because every client comes on his or her own specified day which reduces workload and enables me to have enough time with an individual client”-* Professional nurse, Malawi
	*“[The DSD Models have] lessened the workload. Previously we couldn't spend more time with an individual client but now we do, so thanks to the DSD models we even have a good relationship with the clients.”*—Site operations manager/facility in-charge, Zambia
More time allocated to administrative duties (Maintenance factor: working conditions)	*"[I] consolidate monthly stats and retrieve files for pre-booked clients for the week."*—Admin clerk, South Africa
	*“Now, we are able to look at registers and other administrative duties properly. A long time ago when there were no DSD models, I believe it used to be crowded here. And that meant a lot of us providers didn't have enough time to do other administrative work properly.”*—Professional nurse, Zambia
More time to other ART-related tasks (Maintenance factor: working conditions)	*“I use extra time to write reports and do client tracing.”*—Lay counsellor, Malawi
	*"I go through my daily clients register, checking those who didn't come for their appointments, then I call to remind them about their appointments. If the call is unsuccessful, I do the physical visit."*—Staff nurse, South Africa
More time spent assisting other departments (Maintenance factor: working conditions)	*“I use the extra time to work at antenatal and labour ward.”*—Staff nurse, Malawi
	*"I get to see other clients who are not on DSD models like acute clients."*—Professional nurse, South Africa
Other changes in workload (Maintenance factor: working conditions)	*“The workload is lighter than previously. Stock holding is less. Ordering is more stable than before as previously I would go to sister clinics to borrow what I didn't have as I always ran short due to the influxes of clients.”*—Pharmacy assistant, South Africa

A minority of providers across the three countries said that they worked shorter hours after DSD implementation (24% Malawi, 7% South Africa, 41% Zambia) or that DSD models required them to spend more time on administrative duties (10% Malawi, 22% South Africa, 30% Zambia).

In qualitative results, providers principally described having additional time for routine administrative duties and management tasks, including updating and organizing files, preparing for the next day’s clients, entering client and facility data, writing reports, and conducting audits. Many nurses and some doctors discussed assisting colleagues in other wards/departments, particularly the outpatient department and antenatal and labour wards. Non-clinical providers mentioned providing additional ART client support and tracing, including reviewing registers to track upcoming and missed appointments, calling clients, and conducting additional testing and counselling.

While DSD models reduced clinic congestion, many providers reported being assigned additional administrative duties, supporting client tracing, or assisting other departments in the time that was freed up. When asked about compensation, most providers from South Africa and Zambia did not receive additional compensation (salary or overtime payments for weekends or benefits) as a result of having DSD models at their facilities, but 71% of healthcare providers from Malawi reported receiving extra compensation such as lunch and transport allowances for when they had to travel for DSD.

### Provider job satisfaction

Providers generally reported high levels of job satisfaction related to DSD implementation. 84% of providers from Zambia, 64% from South Africa, and 50% from Malawi had medium to high levels of satisfaction. Providers in all three countries agreed that DSD models made them like their job more, improved how time was spent, and improved relationships with clients. Most reported that DSD implementation did not reduce their working hours. The perception of DSD implementation on providers’ levels of breaks and personal time varied across countries (Malawi: 60%; South Africa: 49%; Zambia: 81%).

**Fig. 2:**
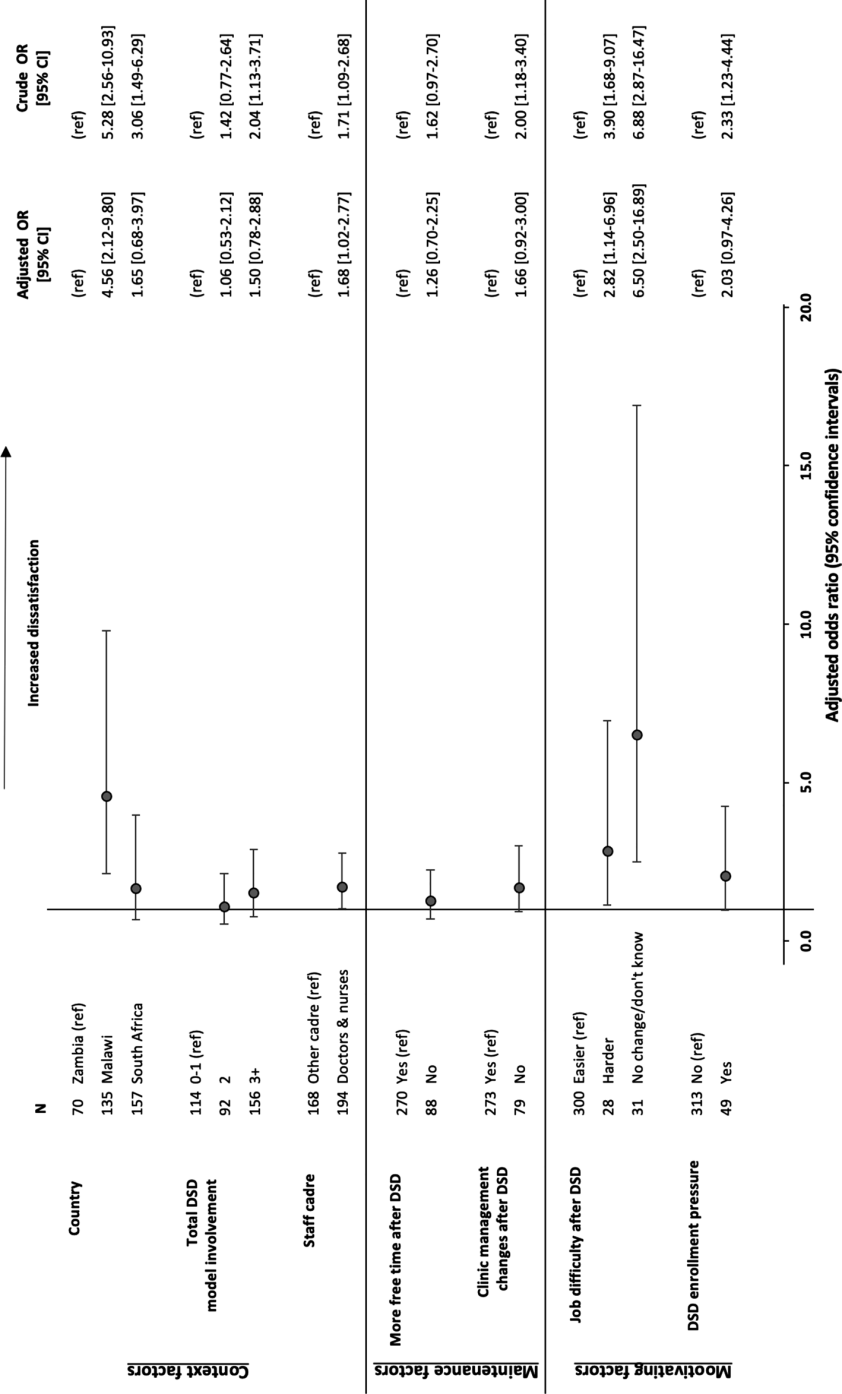
Crude and adjusted odds ratios of factors associated with low job satisfaction among healthcare workers in Malawi, South Africa, and Zambia

Providers from Malawi were more likely to report lower job satisfaction than those from South Africa or Zambia (adjusted odds ratio (aOR) 4.56; 95% CI [2.12–9.80]) (Fig. 2). Providers who believed their jobs became harder after the introduction of the DSD models (2.82; [1.14–6.96]) or their jobs did not change (6.50; [2.50–16.89]) were also more likely to report lower job satisfaction than those who believed their jobs became easier, particularly in South Africa. Those who had responsibilities in three or more DSD models (1.50; [0.78–2.88]) and experienced pressure to enrol clients in DSD models (2.03; [0.97–4.26]) trended toward association with lower job satisfaction.

Nearly all respondents across the countries reported in their open-ended (qualitative) responses that DSD models made their jobs easier. They reported that the decongestion of the clinics resulted in fewer clients managed each day, a reduced workload, less stress, a more organized work environment that allows for better planning and teamwork, and more time for other work (maintenance factors). Providers also described being satisfied with their jobs because they were passionate about helping clients and felt the decanting of clients allowed them to spend more time with each client and provide higher quality care, a motivating factor (Table 5, 6).

**Table 5:** Components of job satisfaction among providers who delivered care prior to DSD model implementation and summary of principal component analysis

	Malawi (*N* = 136)	South Africa (*N* = 158)	Zambia (*N* = 70)
Agree/strongly agree with the following components of job satisfaction, n (%)			
*I like my job more now*	131 (96%)	144 (91%)	69 (99%)
*Clients who are in other models of treatment are more polite or more friendly than those who are not*	66 (49%)	100 (63%)	48 (69%)
*Having other models of treatment delivery available has improved my relationship with clients*	122 (90%)	133 (84%)	69 (99%)
*I am more satisfied with my job than I was*	117 (86%)	116 (73%)	64 (91%)
*Having other models of treatment delivery has made my relationships with colleagues in the facility better*	104 (76%)	129 (82%)	60 (86%)
*Having other models of treatment delivery has made my relationships with senior management better*	96 (71%)	108 (68%)	63 (90%)
*I feel happier overall (in my life) than I used to*	107 (79%)	123 (78%)	68 (97%)
Overall level of satisfaction*, n (%)			
*High*	68 (50.4)	100 (63.7)	59 (84.3)
*Low*	67 (49.6)	57 (36.3)	11 (15.7)

*Overall satisfaction was determined using principal component analysis of the preceding 7 questions: high satisfaction indicates scores greater than 4 and low satisfaction indicates scores less than or equal to 4

**Table 6:** DSD model-based reasons for respondents’ higher/lower job satisfaction, categorized by Maintenance or Motivator

Emerging theme categorized by factors	Illustrative quotes
*Maintenance factors (can cause dissatisfaction if lacking)*	
Decongestion of facilities, more time to reallocate to administration and other tasks (working conditions)	*“DSD models have decongested the facility which has led to my job being more efficient and effective. It has also helped me meet my individual and facility targets, which has led to my Job satisfaction*.”—Facility Manager, Zambia
Better structure and management of staff (working conditions)	*“There is more structure and the role of the staff are more defined, more accountability and even the monitoring are more defined and the report are more accurate”*—Facility Manager, South Africa
	*“… In terms of human resources, the ratio has become proportional, time has also been managed*.”—Professional Nurse, Zambia
*Motivating factors (can influence degree of satisfaction)*	
Feeling pressure to enrol clients on DSD models and high targets to meet (achievement)	*“The pressure to have more clients virally suppressed and enrolled on DSD model.”—*Pharmacist, Zambia
	*“Unrealistic targets that are set” -*Professional Nurse, South Africa
DSD models have made jobs easier, allowed for more time with clients (work itself)	*“My work has become easier now because the DSD's have been categorised …”—*Expert Client, Malawi *“In terms of my work schedule, now my work schedule is not stressful and because of that I spend much time with my clients thereby making me so satisfied.”—*Professional Nurse, Malawi
Predictability of work responsibilities (responsibilities)	*“I know in advance the kind of clients am to see on the particular day so it enables me to prepare”—*Adherence Support Officer, Malawi

Numerous providers discussed the clinic being better managed, organized, and/or more appropriately staffed after the introduction of the DSD models. Multiple providers described the DSD models creating a more organized work environment because they can better plan and know ahead of time which kinds of clients are coming on which days. In addition, a few providers explained that clinic staff have clearer, more definite roles with greater accountability, resulting in improved clinic management.

Respondents who were less motivated explained that they had higher workloads and experienced pressure to meet facility targets, such as achieving viral suppression in clients, enrolling clients into DSD models, and retaining clients in care. They described the targets as being “unrealistic" and explained that their job performance was measured against these targets. These responses were similar among doctors, nurses, and other staff cadres. Some providers also described feeling pressure and/or dissatisfied because of insufficient resources at the facilities (particularly human resources due to staff shortages or absenteeism), the challenges of client adherence, and difficulties with clients not understanding the purpose of DSD models. A higher proportion of South African (23%) nurses reported pressure to enrol clients into DSD models, compared to Malawi (4%) and Zambia (0%) nurses (see Supplementary Table 3).

## Discussion

Healthcare providers’ job satisfaction and perceptions of their workloads have been found to affect the quality of care provided and clients’ treatment outcomes [29, 30], as well as providers' willingness to remain in their positions [17, 31]. In this survey in Malawi, South Africa, and Zambia, providers reported generally higher job satisfaction and a positive change in their workloads after the introduction of differentiated service delivery for HIV treatment.

Our results suggest that, from the perspectives of health workers in Malawi, South Africa and Zambia, the introduction of DSD models primarily affected maintenance factors within Herzberg’s two-factor theory. Most survey respondents indicated that DSD models reduced their workloads and made their jobs easier, and that the decongested clinics resulted in less stress and more time for needier clients and for administrative tasks. Providers attributed the decongestion of clinics to the existence of external dispensing points and multi-month dispensing. Respondents from South Africa were more likely to link their job satisfaction with the improvement in facility management and organization that came with the introduction of DSD models, which allowed for streamlining of clinic services. Across the three countries, providers highlighted fewer ART clients, sufficient space for other waiting clients, and clearer roles for providers resulting in greater accountability as DSD-associated improvements.

DSD models also affected several of Herzberg’s motivating factors. DSD models, via clinic decongestion, allowed providers to spend more time on their clinical work and offered opportunities to pursue more training. In Malawi, respondents linked satisfaction with their job to being able to provide clients with better care and to improvements in provider–client relationships. This finding was very similar to reports from Zambian respondents who linked their satisfaction to being able to offer better quality of care to clients and to spend more time with individual clients. Being able to spend sufficient time with individual clients has been found to be an important contributor to provider satisfaction in other settings [32].

Our findings support the ex ante expectation that the introduction of DSD models will reduce the ratio of client visits per provider, thereby allowing providers to spend more time with clients who are not eligible for DSD models [33]. Our results also add to the existing body of research on self-reported provider workload following DSD implementation in other sub-Saharan African countries. Healthcare workers from Eswatini reported decreased workload, reduced clinic congestion, and shorter client waiting times following the implementation of DSD models [34]. Providers in Mozambique reported an overall improvement in quality of care due to reduced provider workload, which allowed them to attend to more complicated clients [35]. In a small qualitative study in Zambia, most providers preferred dispensing ART medications over a 6-month period rather than 3 months, aligning with clients' clinical appointments. These providers also perceived significant benefits related to reduced workload, ability to see unstable clients, and decongested clinics [36].

Although most responses were positive, a minority of respondents in all three countries stated that the introduction of DSD models reduced their job satisfaction due mainly to additional administrative duties and lack of human and financial resources at their facilities. This was more commonly reported by South African respondents and mirrors findings from studies in Zimbabwe [37] and Eswatini [34], in which providers reported that having to fill prescriptions for community-based models added to their job burdens.

In our study, those who did not perceive any changes in their jobs after DSD implementation were more likely to report lower job satisfaction. To the extent that lack of change reduced satisfaction, it seems likely that providers did anticipate changes, and some were disappointed to find their expectations unmet. Improving the information offered to healthcare providers before DSD adoption may help to limit this outcome. The specific structure of the DSD models in use—for example, whether the models are facility- or community-based and healthcare providers’ responsibilities for them—may also be an important determinant of satisfaction. We also found that those who felt more pressure to enrol clients in DSD models were more likely to report lower job satisfaction. The qualitative results revealed that providers felt differently about the targets to enrol clients in DSD. Some seemed to find this motivating, increasing job satisfaction, and others disliked the pressure, reducing job satisfaction.

Pressure to enrol clients in DSD models is likely coming from both government health agencies and international funders, which can set formal or implied targets for DSD enrolment [38–40]. Whether the pressure perceived by providers improves uptake among eligible clients or instead leads to enrolment of ineligible or unwilling clients is unclear. Concern about under-enrolment of eligible clients in models that have been demonstrated to improve client satisfaction and reduce both client and provider costs is understandable, and enrolment targets may help address this concern. On the other hand, pressure to meet enrolment targets may compromise provider adherence to the principles of client-centred care by not offering choices to clients and/or ignoring clinically appropriate eligibility criteria. It may also mask the benefits of DSD models and cause providers to regard them as burdensome rather than helpful and to influence clients’ own knowledge and perceptions of DSD. While these issues remain speculative, our results indicate that enrolment pressure does reduce job satisfaction for some providers.

Our survey had a number of strengths, including using mixed methods and collecting data from providers at 42 diverse facilities in three countries. Our approach came with important limitations, however. Our total sample size, while larger than in many of the other studies cited, was small and precluded stratification by provider characteristics other than country and staff cadre. Participants were referred to us by facility managers and thus may not be fully representative of all providers. Like all the other studies cited above, moreover, our analysis relied on self-reported responses from participants with regard to both satisfaction and workload. Recall bias is likely, as the original adoption of DSD models occurred at least a year before the survey at most study sites. Satisfaction itself is challenging to measure and compare [30], particularly in a cross-sectional survey, due to its close relationship to individual experiences and expectations and to its potential to vary from day to day, in response to short-term circumstances.

In addition, we did not measure to what extent providers’ actual workload changed as a result of DSD implementation or the uses that were made of any “freed up” time. It is likely that some re-allocation of provider time resulted in the delivery of more or better-quality care, while some was likely used less productively. Quantitative analysis of time-and-motion data, longitudinal ratios of staff to clients before and after DSD scaleup, and data on outcomes of clients not enrolled in DSD models will be needed to confirm self-reports of the value of freed-up time. Finally, the survey was conducted during a period of varying levels of COVID-19 infections and restrictions which may also have affected provider job satisfaction; we did not collect data about the impact of the pandemic.

## Conclusions

DSD models have the potential to improve not only the client but the provider experience. In our study sample, the introduction of DSD models seems predominantly to have improved maintenance factors, particularly working conditions. This change is fundamental to preventing dissatisfaction and is a necessary prerequisite for increasing satisfaction. For most respondents, moreover, DSD models allowed more time with their clients, a motivating factor that is well known to improve provider satisfaction by creating a greater sense of fulfilment and purpose in their work [32]. Future rounds of the SENTINEL study will explore questions of provider satisfaction and workloads over time and the role of ongoing DSD model scale-up in influencing provider satisfaction.

## Supplementary Information

Supplementary material 1: Supplementary Table 1. Characteristics of the SENTINEL study sites. Supplementary Table 2. Survey participants’ roles in DSD models, N=468. Supplementary Table 3. Self-reported effect of DSD model implementation on respondents’ workloads, by professional cadre, N=468.

## Data Availability

All data used in this study were collected by the study team following written informed consent. Data will be made available within one year after the closure of the study by the supervising ethics committees. At that time, data will be posted in a public data repository.

## Abbreviations

SSA: Sub-Saharan Africa
DSD: Differentiated service delivery
ART: Antiretroviral therapy
6MMD: Six-month medication dispensing
CAAPs: Community ART access points
