# Supplementary material for: Effect of differentiated service delivery models for HIV treatment on healthcare providers’ job satisfaction and workloads in sub-Saharan Africa: a mixed methods study from Malawi, Zambia, and South Africa
Source: Hum Resour Health. 2025 May 26;23:25. doi: 10.1186/s12960-025-00993-6 (PMC12105310; doi:10.1186/s12960-025-00993-6)
Supplement: Supplementary file 1 — Supplementary material 1: Supplementary Table 1. Characteristics of the SENTINEL study sites. Supplementary Table 2. Survey participants’ roles in DSD models, N=468. Supplementary Table 3. Self-reported effect of DSD model implementation on respondents’ workloads, by professional cadre, N=468. [file 12960_2025_993_MOESM1_ESM.docx]

### **Supplementary Table 1. Characteristics of the SENTINEL study sites**

| **Site** | **Setting** | **Number on ART 2021** | **% of ART clients enrolled in DSD models (2021)** |
| --- | --- | --- | --- |
| **Malawi** |  |  |  |
| *Blantyre District* |  |  |  |
| Health Centre | Urban | 3,298 | 31% |
| Health Centre | Urban | 9,032 | 97% |
| Health Centre | Urban | 6,295 | 95% |
| Mission Hospital | Rural | 6,473 | 17% |
| *Chiradzulu District* |  |  |  |
| Health Centre | Rural | 2,987 | 90% |
| Health Centre | Rural | 2,871 | 77% |
| District Hospital | Rural | 6,635 | 73% |
| Health Centre | Rural | 4,020 | 92% |
| *Lilongwe District* |  |  |  |
| Health Centre | Rural | 1,221 | 98% |
| Mission Hospital | Rural | 2,646 | 59% |
| Hospital | Urban | 24,895 | 95% |
| Health Centre | Urban | 4,592 | 98% |
| **South Africa** |  |  |  |
| *Ekurhuleni District* |  |  |  |
| Clinic | Urban | 2,386 | 53% |
| Clinic | Urban | 2,658 | 44% |
| Clinic | Urban | 7,213 | 51% |
| *West Rand District* |  |  |  |
| Clinic | Urban | 1,783 | Missing |
| Clinic | Rural | 1,803 | 32% |
| Clinic | Urban | 1,897 | 24% |
| Clinic | Urban | 2,116 | 57% |
| Clinic | Rural | 2,301 | 43% |
| Clinic | Urban | 2,959 | 66% |
| *Ehlanzeni District* |  |  |  |
| Community Health Centre | Urban | 6,622 | 61% |
| Clinic | Rural | 3,553 | 44% |
| Clinic | Rural | 1,943 | 48% |
| Clinic | Rural | 3,001 | 11% |
| Community Health Centre | Urban | 5,234 | 25% |
| Clinic | Urban | 5,515 | 28% |
| *King Cetshwayo District* |  |  |  |
| Clinic | Rural | 1,182 | 24% |
| Clinic | Rural | 1,509 | 81% |
| Clinic | Rural | 2,231 | 78% |
| Clinic | Rural | 3,361 | Missing |
| Clinic | Rural | 5,190 | 61% |
| Clinic | Urban | 7,934 | 75% |
| **Zambia** |  |  |  |
| *Central Province* |  |  |  |
| Mission Health Centre |  | 683 | 85% |
| Urban Health Centre | Urban | 6,985 | 71% |
| Urban Health Centre | Urban | 8,411 | 76% |
| Health Centre | Urban | 3,912 | 95% |
| Mission Hospital | Rural | 3,724 | 85% |
| District Hospital | Rural | 4,326 | 96% |
| *Lusaka Province* |  |  |  |
| Urban Health Centre | Urban | 2,370 | 33% |
| Rural Health Centre | Rural | 4,060 | 89% |
| Health Centre | Rural | 3,754 | 92% |
| 1st Level Hospital | Urban | 11,069 | 77% |
| 1st Level Hospital | Urban | 14,486 | 66% |
| Urban Health Centre | Urban | 6,954 | 81% |

### **Supplementary Table 2. Survey participants’ roles in DSD models, N=468**

| **Variable** | **Malawi, n=142** | | | | **South Africa, n=206** | | | | **Zambia, n=120** | | | |
| --- | --- | --- | --- | --- | --- | --- | --- | --- | --- | --- | --- | --- |
|  | **Nurses** | **Counselors and CHWs** | **Doctors, MOs, COs,** | **Other** | **Nurses** | **Counselors and CHWs** | **Doctors, MOs, COs,** | **Other** | **Nurses** | **Counselors and CHWs** | **Doctors, MOs, COs,** | **Other** |
| N | 49 | 31 | 36 | 26 | 111 | 47 | 1 | 47 | 27 | 45 | 16 | 32 |
| Average days/week spent on all HIV services delivery |  |  |  |  |  |  |  |  |  |  |  |  |
| 0-2 days | 7 (14%) | 1 (3%) | 6 (17%) | 2 (8%) | 15 (14%) | 2 (4%) | 1 (100%) | 6 (13%) | - | 5 (11%) | - | - |
| 2.5-4 days | 12 (24%) | 6 (19%) | 5 (14%) | 3 (11%) | 16 (14%) | 2 (4%) | - | 5 (11%) | 3 (11%) | 3 (7%) | - | 1 (3%) |
| 4.5+ days | 30 (61%) | 24 (78%) | 25 (69%) | 21 (81%) | 80 (72%) | 43 (91%) | - | 36 (77%) | 24 (89%) | 37 (82%) | 16 (100%) | 31 (97%) |
| Average days/week spent on HIV treatment delivery |  |  |  |  |  |  |  |  |  |  |  |  |
| 0-2 days | 11 (22%) | 2 (6%) | 7 (19%) | 3 (12%) | 24 (22%) | 18 (38%) | 1 (100%) | 17 (36%) | - | 5 (11%) | 1 (6%) | 4 (13%) |
| 2.5-4 days | 11 (22%) | 6 (19%) | 6 (17%) | 4 (15%) | 17 (15%) | 3 (6%) | - | 6 (13%) | 4 (15%) | 5 (11%) | 1 (6%) | 1 (3%) |
| 4.5+ days | 27 (55%) | 23 (74%) | 23 (64%) | 19 (73%) | 70 (63%) | 26 (55%) | - | 24 (51%) | 23 (85%) | 35 (78%) | 14 (88%) | 27 (84%) |
| Average days/week spent on DSD model delivery |  |  |  |  |  |  |  |  |  |  |  |  |
| 0-3 days | 23 (47 %) | 13 (42%) | 15 (42%) | 12 (46%) | 34 (31%) | 13 (28%) | - | 14 (30%) | 4 (15%) | 10 (22%) | 3 (19%) | 1 (3%) |
| 4+ days | 26 (53%) | 18 (58%) | 21 (58%) | 14 (54%) | 38 (34%) | 18 (38%) | - | 11 (23%) | 23 (85%) | 34 (76%) | 13 (81%) | 31 (97%) |
| No direct DSD responsibilities | - | - | - | - | 39 (35%) | 16 (34%) | 1 (100%) | 22 (47%) | - | 1 (2%) | - | - |
| Number of DSD models involved in |  |  |  |  |  |  |  |  |  |  |  |  |
| 1 model | 5 (10%) | 4 (13%) | 6 (17%) | 5 (19%) | 25 (23%) | 16 (34%) | - | 8 (17%) | 4 (15%) | 5 (11%) | 2 (13%) | 4 (13%) |
| 2 models | 14 (29%) | 13 (42%) | 7 (19%) | 8 (31%) | 21 (19%) | 13 (28%) | - | 10 (21%) | 7 (26%) | 9 (20%) | 1 (6 %) | 8 (25%) |
| 3+ models | 30 (61%) | 14 (45%) | 23 (64%) | 13 (50%) | 26 (23%) | 2 (4%) | - | 7 (15%) | 16 (59%) | 30 (67%) | 13 (81%) | 20 (63%) |
| No direct DSD responsibilities | - | - | - | - | 39 (35%) | 16 (34%) | 1 (100%) | 22 (47%) | - | 1 (2%) | - | - |

**Admin clerks, data capturers and pharmacists were categorised as other staff cadre.*

*MO, Medical Officer; CO, Clinical Officer; CHW, Community Health Worker*

### **Supplementary Table 3. Self-reported effect of DSD model implementation on respondents’ workloads, by professional cadre, N=468**

| **Variable** | **Malawi, n=142** | | | | **South Africa, n=206** | | | | **Zambia, n=120** | | | |
| --- | --- | --- | --- | --- | --- | --- | --- | --- | --- | --- | --- | --- |
|  | **Nurses** | **Counselors and CHWs** | **Doctors, MOs, COs,** | **Other** | **Nurses** | **Counselors and CHWs** | **Doctors, MOs, COs,** | **Other** | **Nurses** | **Counselors and CHWs** | **Doctors, MOs, COs,** | **Other** |
| NN | 49 | 31 | 36 | 26 | 111 | 47 | 1 | 47 | 27 | 45 | 16 | 32 |
| More free time after DSD (Yes) | 34 (71%) | 23 (74%) | 28 (78%) | 20 (77%) | 72 (66%) | 28 (61%) | 1 (100%) | 37 (80%) | 26 (96%) | 42 (93%) | 15 (94%) | 29 (91%) |
| Different compensation for DSD (benefits, overtime pay) (Yes) | 31 (63%) | 27 (87%) | 27 (75%) | 16 (62%) | 1 (1%) | 7 (15%) | - | 1 (2%) | 2 (7%) | 6 (13%) | 1 (6%) | 3 (9%) |
| DSD enrolment pressure (Yes) | 2 (4%) | 6 (19%) | 1 (3%) | - | 25 (23%) | 6 (13%) | - | 9 (19%) | - | 5 (11%) | 3 (19%) | 2 (6%) |
| Clinic management changes after DSD (Yes) | 37 (77%) | 23 (74%) | 32 (89%) | 21 (81%) | 69 (66%) | 31 (74%) | 1 (100%) | 33 (77%) | 24 (89%) | 40 (93%) | 15 (94%) | 29 (91) |
| Job difficulty after DSD |  |  |  |  |  |  |  |  |  |  |  |  |
| Easier | 45 (92%) | 25 (81%) | 33 (92%) | 25 (96%) | 76 (70%) | 34 (75%) | 1 (100%) | 36 (78%) | 27 (100%) | 43 (98%) | 16 (100%) | 32 (100%) |
| Harder | 3 (6%) | 3 (10%) | 2 (6%) | 1 (4%) | 15 (14%) | 3 (7%) | - | 2 (4%) | - | 1 (2%) | - | - |
| No change/don't know | 1 (2%) | 3 (10%) | 1 (3%) | - | 18 (16 %) | 8 (18%) | - | 8 (17%) | - | - | - | - |
| Do DSD models improve or worsen the care that this facility’s ART clients receive? (Improve) | 49 (100%) | 31 (100%) | 36 (100%) | 26 (100%) | 105 (95%) | 44 (94%) | 1 (100%) | 42 (89%) | 27 (100%) | 45 (100%) | 16 (100%) | 32 (100%) |

**Admin clerks, data capturers and pharmacists were categorised as other staff cadre.*

*MO, Medical Officer; CO, Clinical Officer; CHW, Community Health Worker*
